# Case of Needle-Swallowing

**Published:** 1859-10

**Authors:** 


*Case of Needle-Swallowing.*—A young woman accidentally swallowed, during an inspiration, a sewing needle, which she held between her lips. This occurred September 14th. On December 28th, she drew attention to a small but very painful swelling at the anterior part of the left side of the thorax. In this the needle could be plainly felt. It lay between the sixth and seventh ribs, at a short distance from the lower end of the ensiform process of the sternum. It was extracted by means of an incision from among some strong adhesions by which it was surrounded. For two or three weeks the girl had suffered from a dyspnœa that could not be accounted for; but as this ceased as soon as the needle could be felt under the integuments, Dr. Siegmund, who reports the case, believes that it was produced by the passage of the needle through the diaphragm.—*Virchow Arch.*—*Ibid.*

